# ON the Nature of Ionic Liquid Gating of La_2−x_Sr_x_CuO_4_

**DOI:** 10.3390/ijms19020566

**Published:** 2018-02-13

**Authors:** Hasan Atesci, Wouter Gelling, Francesco Coneri, Hans Hilgenkamp, Jan M. van Ruitenbeek

**Affiliations:** 1 Huygens-Kamerlingh Onnes Laboratorium, Universiteit Leiden, Postbus 9504, 2300 RA Leiden, The Netherlands; atescihasan0@gmail.com (H.A.); wouter.gelling@outlook.com (W.G.); 2 MESA+ Institute for Nanotechnology, University of Twente, P.O. Box 217, 7500 AE Enschede, The Netherlands; francesco.coneri@gmail.com (F.C.); J.W.M.Hilgenkamp@utwente.nl (H.H.)

**Keywords:** La_2−x_Sr_x_CuO_4_, ionic liquid, electric double layer, oxygenation

## Abstract

Ionic liquids have recently been used as means of modulating the charge carrier properties of cuprates. The mechanism behind it, however, is still a matter of debate. In this paper we report experiments on ionic liquid gated ultrathin La_2−x_Sr_x_CuO_4_ films. Our results show that the electrostatic part of gating has limited influence in the conductance of the cuprate in the gate voltage range of 0 to −2 V. A non-electrostatic mechanism takes over for gate voltages below −2 V. This mechanism most likely changes the oxygen concentration of the film. The results presented are in line with previous X-ray based studies on ionic liquid gating induced oxygenation of the cuprate materials YBa_2_Cu_3_O_7−x_ and La_2−x_Sr_x_CuO_4_.

## 1. Introduction

Changing the charge carrier density by means of gating is the crux of today’s technology, where fields of up to 10 MV/cm are used in transistors to change semiconductor properties. Recently, ionic liquids (ILs) have been used in oxide semiconductors for the application of fields of up to two orders of magnitude higher than that for their conventional solid-state counterparts. ILs consist entirely of ions and form Helmholtz electric double layers at the interfaces with the electrodes when a voltage is applied across the IL. One layer consist of anions or cations of the IL, while the other layer comprises the induced charge carriers of the solid. With a ∼1 nm separation within the double layer charge carrier densities of up to $8\times 10^{14}\;{\mathrm{cm}}^{-2}$ are achievable [1], making it possible to induce insulator-to-superconductor [2,3,4] and metal-to-insulator transitions [5,6,7].

This technique is driven by an electrostatic mechanism, and holds for many materials [8,9]. However, when applied to correlated oxide systems [5,10] there is substantial evidence that electrochemical processes related to interstitial oxygen is crucial in the gating process [11]. As for cuprate high temperature superconductors such as YBaCu_3_O_7−x_, several reports suggested that the superconducting transition can be induced by (de)oxygenation of these materials by means of IL gating [12,13], while others suggested an electrostatic mechanism [14]. Dubuis et al. [11] argued that the electric field of the electric double layer drives a redistribution of the oxygen atoms in the basal planes of the cuprate in an electrostatic fashion, while others have suggested an electrochemical process leading to oxygenation of the cuprate [12,13]. In this work, we attempt to address the mechanism of the IL gating of the cuprate material of La_2−x_Sr_x_CuO_4_.

## 2. Results

**Figure 1:**
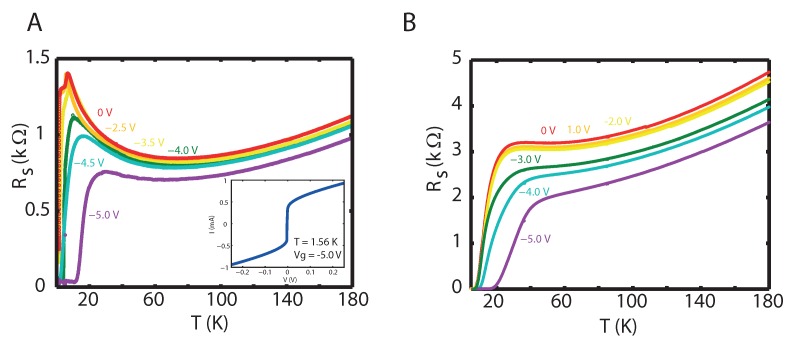
(**A**) Sheet resistance R_S_ plotted against temperature T for various *V*_g_ for a 30 UC thick La_1.95_Sr_0.05_CuO_4_ sample. At *V*_g_ = 0 V, the R(T) characteristic shows that the sample is nearing superconductivity at the lowest T of 1.6 K. This pattern persists for *V*_g_ down to *V*_g_ = −3.5 V. Applying a lower *V*_g_ leads to the emergence of superconductivity with T_c_ growing to 12.0 K when *V*_g_ = −5 V is applied. The inset shows the source-drain current as a function of the voltage over two side contacts at T = 1.6 K and *V*_g_ = −5 V; (**B**) Similar data for a thinner sample of 10 UCs, where the superconducting state is extended in T_c_ only for *V*_g_ below −3 V.

We first present $R(T,V_{g})$ curves for films grown on LaSrAlO_4_. We observe that IL gating induces superconductivity in La_1.95_Sr_0.05_CuO_4_, even when the film has a thickness of 30 unit cells (UC) (Figure 1A). At 0 V, the curve shows the expected characteristic for a Sr doping of 0.05, for which the sample at 1.5 K is at the verge of superconductivity. At gate voltages down to −3.5 V, there is no substantial change with respect to the reference curve. However, when the gate voltage is −4.0 V or below, a superconducting state emerges, with the $T_{c}$ extendable up to 12.0 K (onset) or 19.5 K (midpoint) at $V_{g}=-5$ V. The inset of Figure 1A shows an I(V) curve (T=1.6 K, $V_{g}$ = −5.0 V) having a critical current of approximately 0.3 mA. Thinner samples (10 UC) demonstrate an initial critical temperature $T_{c}$ already before gating which we attribute to the compressive stress exerted on the film (Figure 1B). As is the case for 30 UC films, the reference midpoint $T_{c}$ of 13.5 K is barely altered at gate voltages down to −3 V. The $T_{c}$ can only be increased with sufficiently large negative gate voltages of −4 V or lower, where the lowest gate voltage of −5 V results in a superconducting midpoint temperature of 30.4 K.

**Figure 2:**
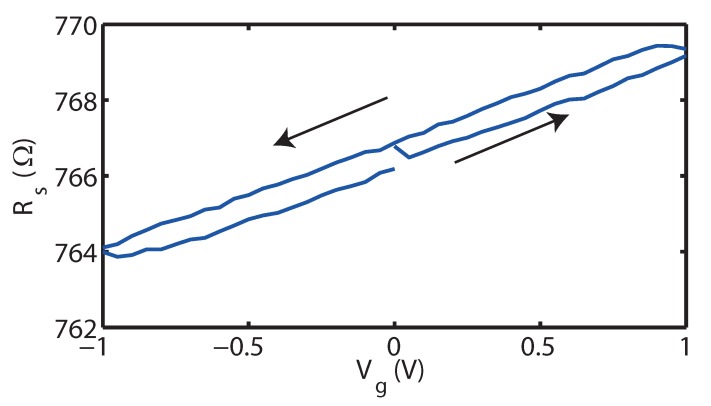
Typical sheet resistance R_S_ plotted against gate voltage *V*_g_ for a 30 UC La_1.91_Sr_0.09_CuO_4_ film grown on (LaAlO_3_)_0.3_(Sr_2_AlTaO_6_)_0.7_. In the given *V*_g_ range, hysteresis is absent (apart from a small instrumental time delay) and we observe a linear relationship having a positive slope, coinciding with hole (electron) doping for negative (positive) gate voltages. The sweep rate is 50 mV/s, while the polarization temperature is 225 K. The arrows indicate the direction of the evolution of the curves in time.

A first step towards investigation of the gating mechanism involves measuring the sheet resistance $R(V_{g})$ characteristics, as is shown for a typical 30 UC La_1.91_Sr_0.09_CuO_4_ film grown on (LaAlO_3_)_0.3_(Sr_2_AlTaO_6_)_0.7_, in Figure 2. We make two main observations on the charging loop, namely the absence of hysteresis (apart from a small instrumental time delay) and a linear response of the resistance of the film as a function of gate voltage. The first observation is characteristic of an electrostatic mechanism, as has been observed before, e.g., in IL-gated indium tin oxide films [11]. This is also consistent with the linear response of the resistance. We can quantify the effects of gating by assuming that the sample can be thought of as having a top and bottom layer. The latter is not affected by gating and has a fixed charge carrier density $n_{0}$. The top layer is in contact with the IL and has a charge carrier density $n_{t}$ which is $V_{g}$ dependent, and we expect this dependence to be linear, $n_{t}\left(V_{g}\right)=n_{0}-\beta V_{g}$. This relationship has a negative slope due to the fact that La_2−x_Sr_x_CuO_4_ is a hole-doped material. The proportionality constant β is positive and is proportional to the gate efficiency between the IL and the top layer. Here, $\beta =\eta \frac{C_{\mathrm{EDL}}}{ed_{t}}$, where η is the electrostatic gate efficiency, while $C_{\mathrm{EDL}}$ is the specific capacitance of the interface to DEME-TFSI, *e* is the electron charge and $d_{t}$ is the top layer thickness. Under ideal circumstances, η=1. Since the observed resistance change is small we take the resistance to be proportional to the charge density and linearize around $V_{g}=0$, which results in $R\left(V_{g}\right)=R_{0}\left(1+\frac{\beta d_{t}}{n_{0}d}V_{g}\right)$. Specifically, $R_{0}$ is the value of the resistance of the La_1.91_Sr_0.09_CuO_4_ channel without applying a gate voltage. Using the slope of the curve, $\frac{\Delta R}{\Delta V_{g}}≃2.4\;\Omega /V$, we obtain an indication of the gate efficiency η, defined as $\eta =\frac{\Delta R}{\Delta V_{g}}\frac{en_{0}d}{R_{0}C_{\mathrm{EDL}}}$. The sample thickness corresponding to the curve in Figure 2 is 30 UCs (50 nm), while the carrier density $n_{0}=9.6\times 10^{20}\;{\mathrm{cm}}^{-3}$ [15] and $C_{\mathrm{EDL}}=13\;\mu F{\mathrm{cm}}^{-2}$ [16], resulting in η=0.15. In other words, while the IL gating is electrostatic in this range, the efficiency of gating is only at about one seventh of its optimal efficiency. This means that a large part of the charge build-up in the electric double layer does not translate to conductivity. We suspect that this is due to the finer details of crystalline quality of the La_2−x_Sr_x_CuO_4_ film, such as the roughness and presence of grain boundaries. This is supported by reports of IL gating on different crystalline qualities of FeSe films in [17]. Here, the authors state that an improved crystallinity and an atomically flat surface can lead to a more optimal charge transfer during the gating process. In a preliminary series of experiments we found that the smoothness of the film surface can be improved following the recipe by Bollinger et al. [18]. By introducing a 1 UC buffer layer of nominally metallic La_1.70_Sr_0.30_CuO_4_ films of La_2−x_Sr_x_CuO_4_ with thicknesses of up to 7 UC show an improved smoothness of the surface in the AFM images, and a substantially higher gating efficiency up to η=0.65.

**Figure 3:**
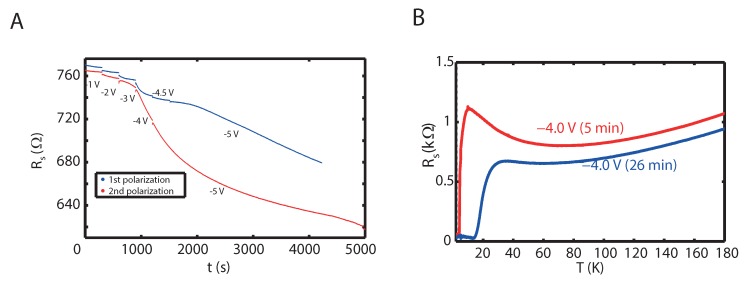
(**A**) Evolution of the sheet resistance $R_{S}$ of a 30 UC La_1.91_Sr_0.09_CuO_4_ film grown on (LaAlO_3_)_0.3_(Sr_2_AlTaO_6_)_0.7_ as a function of time, while the gate voltage is switched to increasing negative values at the points indicated; (**B**) Temperature dependence of the sheet resistance R_S_ for different polarization times and for the first and second polarization at $V_{g}=-4$ V. The midpoint T_c_ increases from 5 K to 17 K for polarization times of 5 and 26 min, respectively.

The gating mechanism was investigated further by altering the gate voltage in steps, while measuring the sheet resistance of a 30 UC La_1.91_Sr_0.09_CuO_4_ film grown on (LaAlO_3_)_0.3_(Sr_2_AlTaO_6_)_0.7_ as a function of polarization time, typical results of which are illustrated in Figure 3A. Two processes are observed upon switching the gate voltage in a step-wise fashion to higher negative values: (1) a nearly instantaneous process which results in the drop of the sheet resistance of the film, and (2) a relatively slow process, requiring several hours to stabilize, becoming most pronounced in the gate voltage regime of −3 V and lower. The first process requires less than 1 s and only produces a limited drop in sheet resistance of ∼2 Ω/V for the gate voltages used, which agrees with the rate found on the basis of Figure 2. The second process only becomes prominent at gate voltages below −3 V, and produces a very pronounced effect in the sheet resistance of the film of up to hundreds of Ω.

Both processes are nearly reversible. After returning to zero gate potential the sheet resistance relaxes to within a percent of the initial value. However, a new cycle of increasing steps of negative gate potential shows that the slow process has become more active. The enhancement of the slow process continues in subsequent cycles, but the changes become smaller and the lowest attained resistance saturates. Figure 3B shows that the slow decrease of the resistance is associated with a gradual shift of the superconducting transition temperature, confirming that it involves a process of hole doping of the copper oxide layer. For higher negative gate potentials at longer polarization times the resistance starts to increase, leading to an irreversible deterioration of the film.

## 3. Discussion

At the polarization temperature at which these measurements were performed (225 K) the build-up time of the electric double layer on the channel should occur within an RC time determined as $R_{\mathrm{IL}}C_{\mathrm{EDL}}$. Here, $R_{\mathrm{IL}}$ is the electrical resistance of the IL, which is of the order of $10^{8}\;\Omega$ [19]. The electric double layer capacitance $C_{\mathrm{EDL}}$ is determined by the specific capacitance of the IL (13 $\mu F{\mathrm{cm}}^{-2}$) and area of the La_2−x_Sr_x_CuO_4_ channel (300 × 50 $\mu m^{2}$), giving $C_{\mathrm{EDL}}≃2$ nF. The estimated RC time becomes of the order of a second. Hence, we conclude that the relatively quick process in the resistance behavior is the contribution of the electrostatic mechanism of gating. The second, much slower process does not behave according to this electrostatic RC time.

Some processes, which can be capacitive or Faradaic in nature, tend to be considerably slower compared to the EDL charging time. These are often related to reconstruction phenomena and ordering effects of the ions of the EDL [20,21]. Furthermore, the processes of this origin tend to become extremely slow at the used charging temperatures [19], many orders of magnitude slower than what is observed in our experiments. We therefore conclude that the observed slow process involves another, non-electrostatic mechanism.

Since the Sr doping of the film is constant, the change in $T_{c}$ could be caused by an increased interstitial oxygen doping of the film, as interstitial oxygen is well-known for its role in changing the charge carrier density in the CuO_2_ planes and hence superconductive properties of La_2−x_Sr_x_CuO_4_ [22,23]. This behavior has been reported before in the IL-gating of YBa_2_Cu_3_O_7−x_ films [12,24], where it was interpreted as an electric field driven slow redistribution process of the oxygen atoms in the copper oxide planes of the film [13,24].

We observe a crossover at around −2 V, where both components have the same contribution in resistance change. For more negative gate voltages the non-electrostatic component starts to dominate. The non-electrostatic component can lead to a substantial change of the onset of $T_{c}$ (13.7 to 25.0 K) as a function of the polarization time at a constant gate voltage of −4 V (Figure 3B). In case only electrostatics would be at play in the gating of La_2−x_Sr_x_CuO_4_, no change in $T_{c}$ is expected, as the polarization time for both curves is beyond the RC time of the system.

**Figure 4:**
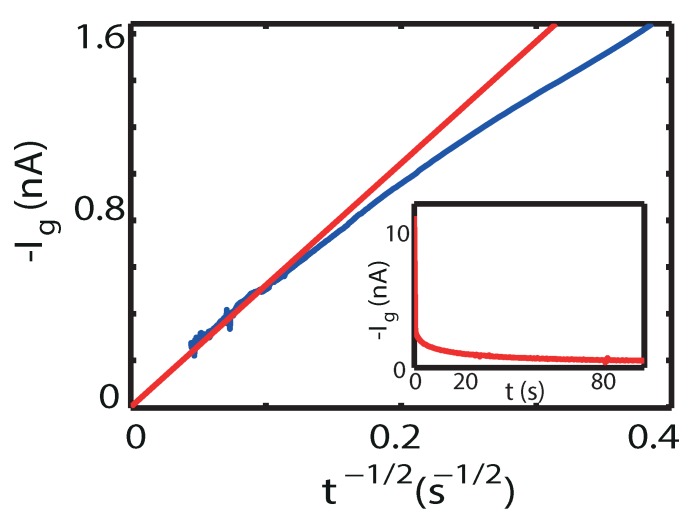
Plot of the gate current *I*_g_ against against $t^{-\frac{1}{2}}$. The behavior follows the Cottrell relationship for diffusion-limited electrochemistry. Inset: −*I*_g_ vs. t. The polarization temperature is 210 K at $V_{g}=-4$ V.

The interpretation of the slow process as an ionic process is further supported by $I_{g}\left(t\right)$ measurements, a typical example of which is shown in Figure 4, taken at $V_{g}\leq -3$ V. In the case of a diffusion-limited charge transfer processes it is known that the gate current under potentiostatic conditions varies according to the Cottrell equation $I_{g}\left(t\right)=FcA\sqrt{D}/\sqrt{\pi t}$. Here, *F* is Faraday′s constant, while *c* is the concentration of the electroactive species, *A* is the electrode area and *D* is the diffusion constant of the particles. As observed in Figure 4, after an initial fast process the gate current eventually follows the $t^{-\frac{1}{2}}$ dependence of the Cottrell equation, the slope of which is 5.2 × 10^−9^ As^−1/2^. Let us assume that the added oxygen is provided from the lower layers of the film. The concentration in the active top layer after a given time can be estimated from the observed optimal $T_{c}=25$ K (onset) at this gate potential of $V_{g}=-4$ V. Using the known variation of $T_{c}$ with hole doping for this compound the oxygen content is estimated as δ≈0.10 [22]. Using the density of the cuprate material, *c* then becomes approximately $1.7\times 10^{-3}$ mol ${\mathrm{cm}}^{-3}$. Accordingly, through the Cottrell equation we obtain a number for the diffusion constant, $D=1.6\times 10^{-14}$ ${\mathrm{cm}}^{2}$ s, which is similar to values known in the literature for electrochemical oxidation of La_2_CuO_4_ at room temperature [25,26]. Alternatively, when assuming that the added oxygen is added from outside the film, i.e., from the ionic liquid, *c* becomes very small, $c∼1\times 10^{-14}$ mol ${\mathrm{cm}}^{-3}$, since the partial pressure of oxygen in the IL is limited by high vacuum conditions. As a result the diffusion constant becomes anomalously high, $D∼1\times 10^{2}$ ${\mathrm{cm}}^{2}$ s, inconsistent with the viscous state at the used polarization temperatures near the glass transition.

Other electrochemical processes can cause a change in the doping of a material. For example, hydrogen doping is known in La_2_CuO_4_, YBa_2_Cu_3_O_7_ [27], and other oxides such as VO_2_ [28]. Protonation is also reported in TiO_2_ [29] and WO_3_ [30]. Although trace amounts of both hydrogen and protons can be present in the IL, both processes are unlikely to happen. Protonation would diminish the hole density of the film and thus decrease the $T_{c}$, which is not observed, whereas hydrogenation would also decrease the $T_{c}$ for the Sr doping used in this study [27].

In summary, our results emphasize the role of electrostatic and non-electrostatic processes in different gate voltage regimes and different crystalline qualities of La_2−x_Sr_x_CuO_4_ films. Compared to the IL-gating of the band insulator SrTiO_3_, for example, the electrostatic mechanism has a negligible effect in the total charge induction in the films, amounting to less than 15%. We suspect that this small electrostatic contribution is primarily controlled by the surface crystalline quality parameters such as surface roughness and presence of grain boundaries. The non-electrostatic process is significantly slower than the expected RC times of the system. From this we conclude that this process most likely involves oxygen doping of the film. The diffusion constants extracted for this process support an interpretation in terms of the oxygen being provided from the lower layers of the film, rather than from the outside. These results are in line with previous IL gating and X-ray absorption measurements [11,13], and will be important in forming a proper understanding for further exploration of this rapidly developing field.

## 4. Materials and Method

**Figure 5:**
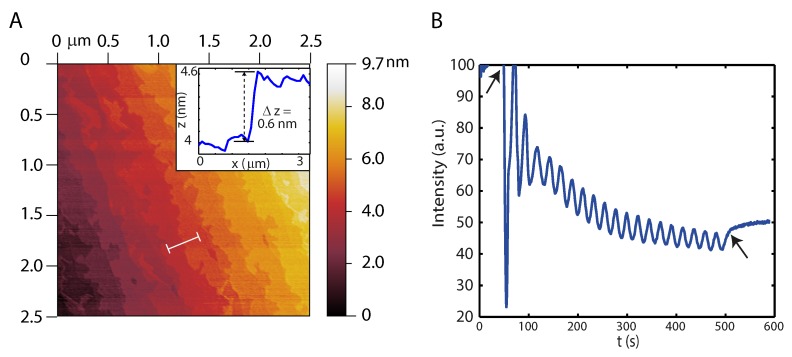
(**A**) Example of an atomic force microscopy (AFM) image of an annealed LaSrAlO_4_ (001) substrate (1050 C, 10 h) showing clear steps. The inset shows the height profile of one step, indicating the step height is half a UC. This is due to the double termination of the substrate UC. The rms roughness is 0.23 nm; (**B**) Graph of Refractive High Energy Electron Diffraction (RHEED) intensity vs. time which shows the oscillations of the specular reflected beam for the growth of a 10 UC thick La_1.95_Sr_0.05_CuO_4_ (Sr doping is 0.05) on LaSrAlO_4_. The oscillations are typically preserved up to the 20th RHEED oscillation and are indicative of layer-by-layer growth of the cuprate. The arrows indicate the start and end of the deposition process.

The La_2−x_Sr_x_CuO_4_ films are grown on single crystalline (LaAlO_3_)_0.3_(Sr_2_AlTaO_6_)_0.7_ or LaSrAlO_4_ (001) substrates (0.05–0.3° miscut), for these have a small mismatch in the lattice constants with respect to La_2−x_Sr_x_CuO_4_ (0.5%). The substrates were annealed at a temperature of 1050 °C for 10 or 12 h under a flow of O_2_ of 50 mL/min to remove any organic materials on the surface of the substrate and to allow reconstruction of surface defects. When scanned with atomic force microscopy (AFM), the substrate surface shows clear steps of half a UC height (Figure 5A), which is expected for a double termination of the substrate lattice, and has a root mean square (rms) roughness of 0.23 nm.

**Figure 6:**
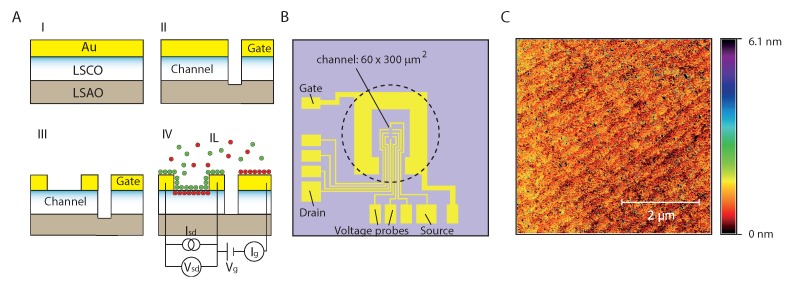
(**A**) Schematics of the sample preparation. (**I**) After in-situ pulsed laser deposition of 10 to 30 UC of La_2−x_Sr_x_CuO_4_ (LSCO) on a LaSrAlO_4_ (LSAO) substrate, followed by Au deposition; (**II**) After dry etching of the areas defined by photolithography; (**III**) After wet etching of the excess Au between the source and drain electrodes using a KI/I_2_/H_2_O solution; (**IV**) Schematic representation of the electronic circuit of the IL/La_2−x_Sr_x_CuO_4_ system; (**B**) Layout of the electrodes and the channel (60 × 300 μm^2^). All yellow colored structures are Au covered, while at nearly all of the violet areas the dry etching process has exposed the substrate, except in the center of the structure between the contacts, where the LSCO channel is uncovered by the wet etching step in KI/I_2_. The dashed circle represents the approximate area covered by the IL droplet at the sample surface; (**C**) An example of an AFM image of the surface of the La_2−x_Sr_x_CuO_4_ channel (10 UC) after fabrication, before adding the IL. The film rms roughness is 0.49 nm, and clear steps of the film are visible.

We use Pulsed Laser Deposition combined with Refractive High Energy Electron Diffraction (RHEED) to grow the cuprate and monitor the growth in-situ. During growth the substrates are kept at a deposition temperature of 740 °C and O_2_ pressure of 0.13 mbar. The target is ablated using a laser fluence of ∼1.65 J/cm^−2^ and repetition rate of 4 Hz. The RHEED oscillations, as seen in Figure 5B, indicate layer-by-layer growth, where two RHEED oscillation periods indicate the growth of 1 UC [14,31]. The thickness of the grown La_2−x_Sr_x_CuO_4_ film is between 10 and 30 UCs, while x varies from 0.05 to 0.09. When Au for the electrodes is deposited ex-situ by a photolithographic process the adhesion of the Au electrodes is poor and the Au layers detach with the lift-off step. To prevent this we have attempted to introduce a thin Ti adhesion layer before depositing Au. However, this introduces high contact resistances, about 100–1000 times the sheet resistance and further diverging at low temperatures, rendering measurements at low temperatures problematic. We suspect that the deposition of Ti leads to oxidization, inducing oxygen diffusion from the La_2−x_Sr_x_CuO_4_ layer to the Ti layer. In turn, this leads to a decrease of charge carriers, which is detrimental for the conductive properties. Another reason may be the difference in work function of Ti (4.33 eV) and La_2−x_Sr_x_CuO_4_ (5.02–5.23 eV [32]), leading to a Schottky-like barrier. The problem was resolved by in-situ deposition of a full Au layer with a typical thicknesses of 50–90 nm immediately following La_2−x_Sr_x_CuO_4_ deposition, at an Ar pressure of 2 × $10^{-2}$ mbar, using a laser fluence of ∼4–4.5 J/cm^2^ and a repetition rate of 4 Hz, see Figure 6(AI). The second process involves a photolithographic step in which areas are defined separating the gate from the active device area, followed by an dry etching step in an Ar^+^ ion beam at 500 kV, see Figure 6(AII). During this step, the areas for the Au electrodes needed for measurement and wire bonding, together with the La_2−x_Sr_x_CuO_4_ channel area are protected by the photoresist. Exposing the channel area requires a third process, illustrated in Figure 6(AIII), in which the Au on top of the channel is etched away selectively using a KI/I_2_/H_2_O solution (mass ratio 4:1:40). We find that this wet etching process preserves the quality of the La_2−x_Sr_x_CuO_4_ film and surface.

In most cases, processes I, II and III leave behind photoresist residuals on the La_2−x_Sr_x_CuO_4_ surface. To remove these residuals, we have attempted to etch the samples in an oxygen plasma (13–16 W, 100 mTorr). However, we find that this process modifies the properties of the cuprate film, inducing an n-type field effect transistor-like behavior upon IL gating. In other words, the conduction increases at positive gate voltages and decreases at negative gate voltages, while the opposite, p-type transistor behavior is expected for La_2−x_Sr_x_CuO_4_. This problem can be circumvented by gently cleaning the surface using an ethanol wetted lens tissue, resulting in an atomically clean surface of the La_2−x_Sr_x_CuO_4_, as is shown in Figure 6C.

A typical layout of the measurement circuit of the La_2−x_Sr_x_CuO_4_ with ionic liquid is shown in Figure 6(AIV). The sample chips are electrically wire bonded to a sample holder of the cryogenic insert. We have used two Keithley 2450 SourceMeters for the experiments, one of which is used to apply a gate voltage $V_{g}$ between the gate and drain electrodes, while simultaneously measuring the gate current $I_{g}$. This arrangement is used for cyclic voltammetry, i.e., measuring the gate current $I_{g}$ as a function of $V_{g}$ in order to check for the presence of Faradeic processes characterized by charge transfer peaks. The gate voltages used in the experiment described in this work lies between −5.5 V and 0 V. The other Keithley SourceMeter is used for setting up an excitation current of 1 μA between the source and drain electrodes, while measuring the four-terminal resistance over the voltage leads. The La_2−x_Sr_x_CuO_4_ channel has dimensions of 60 × 300 μm^2^ and the voltage lead separation is identical to the channel width (see Figure 6B), leading to a simple conversion factor for obtaining the sheet resistance. The gate electrode surface area exposed to the IL is approximately hundred times the combined area of the channel and electrodes (voltage probes, source and drain) in contact with the IL, such that the gate voltage dominantly falls over the interface of the IL with the LSCO channel.

Before any usage of the IL for experiments, the bottle containing the liquid, i.e., *N*,*N*-diethyl- *N*-(2-methoxyethyl)-*N*-methylammonium bis(trifluoromethylsulphonyl)-imide (DEME-TFSI, IoLiTech, 99%), is heated at 60 °C overnight and then permanently stored in a N_2_ glovebox (≤0.1 ppm O_2_/H_2_O). Without further precautions we observe evidence for electrochemical processes from peaks in the cyclic voltammogram. We find that degassing the IL to pressures of 10^−1^ mbar for 15 min suffices to completely remove the Faradeic peaks within the electrochemical window under ambient conditions. In view of our experience with other oxide surfaces we used lower vacuum pressures, down to 10^−6^ mbar, [33] in order to completely suppress electrochemical processes due to water or oxygen contaminants in the IL. In order to extend our gating voltage range beyond the electrochemical window of the IL we lower the temperature at which the gating is performed (charging temperature), following Ref. [1]. In our experiments, the charging temperature was between 210–225 K, which is as low as possible while still remaining above the glass transition of 183 K of the IL. At these temperatures we can apply a gate voltage as far negative as −5.5 V without device degradation and dielectric breakdown, linked to the decreased electrochemical activity of the IL. The typical polarization times used in this work are 15 min.
